# Mechanical Properties of Additively Manufactured Thick Honeycombs

**DOI:** 10.3390/ma9080613

**Published:** 2016-07-23

**Authors:** Reza Hedayati, Mojtaba Sadighi, Mohammad Mohammadi Aghdam, Amir Abbas Zadpoor

**Affiliations:** 1 Department of Mechanical Engineering, Amirkabir University of Technology (Tehran Polytechnic), Hafez Ave, Tehran 158754413, Iran; mojtaba@aut.ac.ir (M.S.); aghdam@aut.ac.ir (M.M.A.); 2 Department of Biomechanical Engineering, Faculty of Mechanical, Maritime, and Materials Engineering, Delft University of Technology (TU Delft), Mekelweg 2, Delft 2628 CD, The Netherlands; a.a.zadpoor@tudelft.nl

**Keywords:** cellular structures, 3D printing, elastic properties, hexagonal honeycomb

## Abstract

Honeycombs resemble the structure of a number of natural and biological materials such as cancellous bone, wood, and cork. Thick honeycomb could be also used for energy absorption applications. Moreover, studying the mechanical behavior of honeycombs under in-plane loading could help understanding the mechanical behavior of more complex 3D tessellated structures such as porous biomaterials. In this paper, we study the mechanical behavior of thick honeycombs made using additive manufacturing techniques that allow for fabrication of honeycombs with arbitrary and precisely controlled thickness. Thick honeycombs with different wall thicknesses were produced from polylactic acid (PLA) using fused deposition modelling, i.e., an additive manufacturing technique. The samples were mechanically tested in-plane under compression to determine their mechanical properties. We also obtained exact analytical solutions for the stiffness matrix of thick hexagonal honeycombs using both Euler-Bernoulli and Timoshenko beam theories. The stiffness matrix was then used to derive analytical relationships that describe the elastic modulus, yield stress, and Poisson’s ratio of thick honeycombs. Finite element models were also built for computational analysis of the mechanical behavior of thick honeycombs under compression. The mechanical properties obtained using our analytical relationships were compared with experimental observations and computational results as well as with analytical solutions available in the literature. It was found that the analytical solutions presented here are in good agreement with experimental and computational results even for very thick honeycombs, whereas the analytical solutions available in the literature show a large deviation from experimental observation, computational results, and our analytical solutions.

## 1. Introduction

Bone has the intrinsic ability of self-healing in the case of being damaged in small areas. However, in large bony defects, bone loses its ability to repair itself by regeneration of new bony tissue. While autologous bone grafting is known as the gold standard in orthopaedic surgery, it has some drawbacks such as limited bone stack and donor site mordibility [1]. Recently, porous titanium and titanium alloy scaffolds have been thoroughly investigated due to their excellent biocompatibility and corrosion resistance, low stiffness (which is necessary for avoiding stress shielding), and good bone regeneration performance.

Additive manufacturing techniques have made it possible to fabricate scaffolds with precisely controlled micro-architecture. Therefore, several 3D unit cell types have been suggested and tested mechanically and biologically when used as the micro-architecture of bone replacing scaffolds [2,3]. Singh showed that in some areas (where bone is very dense), cancellous bone microarchitecture resembles that of a hexagonal honeycomb with thick cell walls [4]. Hexagonal honeycombs or honeycomb-like structures have been manufactured as biomedical implants using different additive manufacturing techniques [5,6]. Recently, various cells have been successfully cultured in collagen scaffold honeycombs [7,8,9]. It is important to study the mechanical behavior of thick honeycomb-like structures, because the mechanical properties of the biomaterials used for tissue regeneration could significantly influence the tissue regeneration performance of biomaterials.

The mechanical properties of honeycombs (such as their stiffness and yield stress) in out-of-plane direction are usually much higher compared to their in-plane properties. This is why more studies have been devoted to the investigation of out-of-plane properties of honeycombs compared to their in-plane properties. However, honeycombs are loaded in-plane in a number of natural structures such as cancellous bone, wood, and cork [10]. Moreover, thick honeycombs could be also useful for energy absorption purposes in the in-plane direction. In this case, the mechanism dominating the structure deformation is bending of the cell walls (rather than sudden buckling of the walls which is common in the out-of-plane deformations of honeycombs) and then face-to face crushing of the cell walls. This can lead to a smoother stress-strain curve with higher energy absorption capacity as compared to the out-of-plane direction, if the geometrical parameters are chosen properly.

Several experimental works have been carried out on the in-plane deformation of honeycombs. Foo et al. [11] obtained linear elastic mechanical properties of Nomex™ honeycomb structures using extensive test programs and compared their results with those of their finite element (FE) models. They observed size effects for the moduli of Nomex™ honeycombs. Papka and Kyriakides [12] compressed honeycomb specimens between stiff flat grips experimentally and numerically. They found out that although the crushing patterns developed during the plateau regime differs from specimen to specimen (caused by specimen size and geometric imperfections), the cell failure mechanism is similar for all cases. Other experimental works on the in-plane mechanical behavior of honeycombs can be found in [13,14,15,16,17]. Several numerical studies have also been carried out using FE methods the most important of which are in the following references [18,19,20].

El-Sayed et al. [21] published the first analytical study on the in-plane mechanical properties of hexagonal honeycombs in which the elastic mechanical properties of a composite under in-plane axial and out-of-plane bending loads were studied, and the plastic deformation properties under in-plane axial loading were characterized. However, as stated in [10], their work had ‘numerous small errors’ which rendered the results unreliable. In 1982, Gibson et al. [10] presented improved analytical relationships for the mechanical properties of hexagonal honeycombs ($E_{1}$, $E_{2},\;\upsilon_{12}$, $\upsilon_{21}$, $\sigma_{y1}$, $\sigma_{y2}$, G, and $\sigma_{el}$). Their results showed good agreement with their experimental results for both rubber and metal honeycombs but only for very small values of wall thickness and relative density.

Masters and Evans [22] developed an analytical model for prediction of elastic constants of honeycombs by decomposing the general deformation of a honeycomb cell into flexural, stretching, and hinging parts. For each of the three mechanisms, force constants were defined while relationships for $E_{1}$, $E_{2},\;\upsilon_{12}$, $\upsilon_{21}$, and G were calculated. The elastic constants calculated from each mechanism were then superimposed to give a general model [23]. Masters and Evans [22] did not obtain any relationship for yield stress. Goswami [24] derived analytical formulas for the elastic properties of hexagonal honeycomb cores. Elemental beam theory was used for each component inside the unit cell to give different elastic properties by means of strain energy concept. The results of their model coincided with the results reported in [10]. Balawi and Abot [25] presented a modified model for commercial hexagonal honeycombs having double wall thickness in vertical walls and some curvature in the neighborhood of cell vertices caused by expansion or corrugation processes during manufacturing.

In all of the above-mentioned works, the Euler-Bernoulli beam theory is the theoretical basis for deriving the analytical relationships. The analytical solutions obtained using the Euler-Bernoulli beam theory are not applicable to thick honeycombs, because a number of simplifying assumptions are used in that theory. It is therefore important to use the Timoshenko beam theory for deriving the analytical relationships that are used for thicker honeycombs (which can be good candidates for replacing dense cancellous bones). In this paper, the stiffness matrix of hexagonal honeycomb structures is obtained by which the elastic properties of honeycomb structures including the elastic modulus, Poisson’s ratio, and yield stress in both major in-plane directions are found. The results obtained from the derived formulas are compared with existing analytical formulas presented by Gibson and Ashby [10] and Masters and Evans [22] as well as to the experimental results of the study of Gibson and Ashby [10] on low density honeycombs, and with the mechanical properties measured for additively manufactured dense honeycombs in this study. Moreover, FE models are created to validate the proposed analytical relationships and to present the steps required for development of a trustworthy numerical tool for investigation of thick honeycomb structures.

## 2. Materials and Methods

### 2.1. Experimental Tests

**Figure 1:**
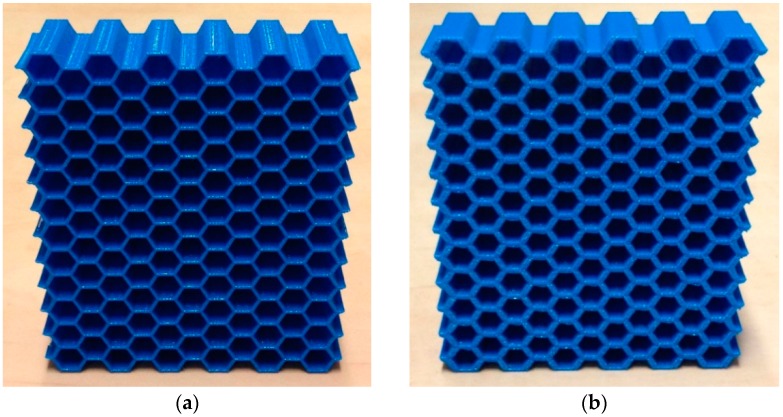

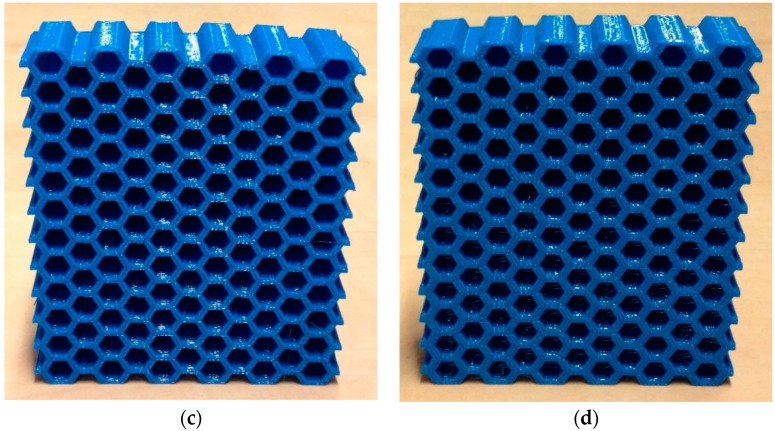
The manufactured hexagonal honeycombs with (**a**) *t*/*l* = 0.09; (**b**) *t*/*l* = 0.18; (**c**) *t*/*l* = 0.27; and (**d**) *t*/*l* = 0.36.

An additive manufacturing technique, i.e., fused deposition modelling, was used for fabricating thick honeycombs with a wide range of relative densities from polylactic acid (PLA). The hexagonal honeycombs were made from poly-lactic acid (PLA) filaments using 5th generation Replicator Desktop Makerbot 3D printer. For each density, six samples were made (three sample for testing in each of the two main directions of each honeycomb). The dimensions of the hexagonal honeycombs were $77\times 90\times 21.395\;{\text{cm}}^{3}$. Four different relative densities of honeycombs were generated by varying the thickness to length ratio of the cell walls, i.e., t/l=0.09, t/l=0.18, t/l=0.27, and t/l=0.36 (Figure 1). The mechanical properties of the samples were measured under compression using INSTRON 5985 machine (Illinois Tool Works Inc., Glenview, IL, USA) with 100 kN load cells. The displacement rate of the upper grip was set to 2 mm/min. The tests and elastic properties calculations were in accordance with the specifications described in the standard ISO 13314:2011 [26]. To measure the mechanical properties of the bulk material, i.e., additively manufactured PLA used in the current study, bulk cylinders (100% infill) with nominal diameters of 12.7 mm and nominal lengths of 25.4 mm were made and tested under compression using a methodology similar to that of the honeycombs. The measured elastic modulus and yield stress of the bulk material were 1.962 ± 0.069 GPa and 56.204 ± 1.213 MPa, respectively. To gain a better overview of the elastic modulus and yield stress values, their normalized values (i.e., ratio of their value in the porous structure to their corresponding value in the bulk material) were plotted and compared between the analytical, numerical, and experimental values.

### 2.2. Relative Density

**Figure 2:**
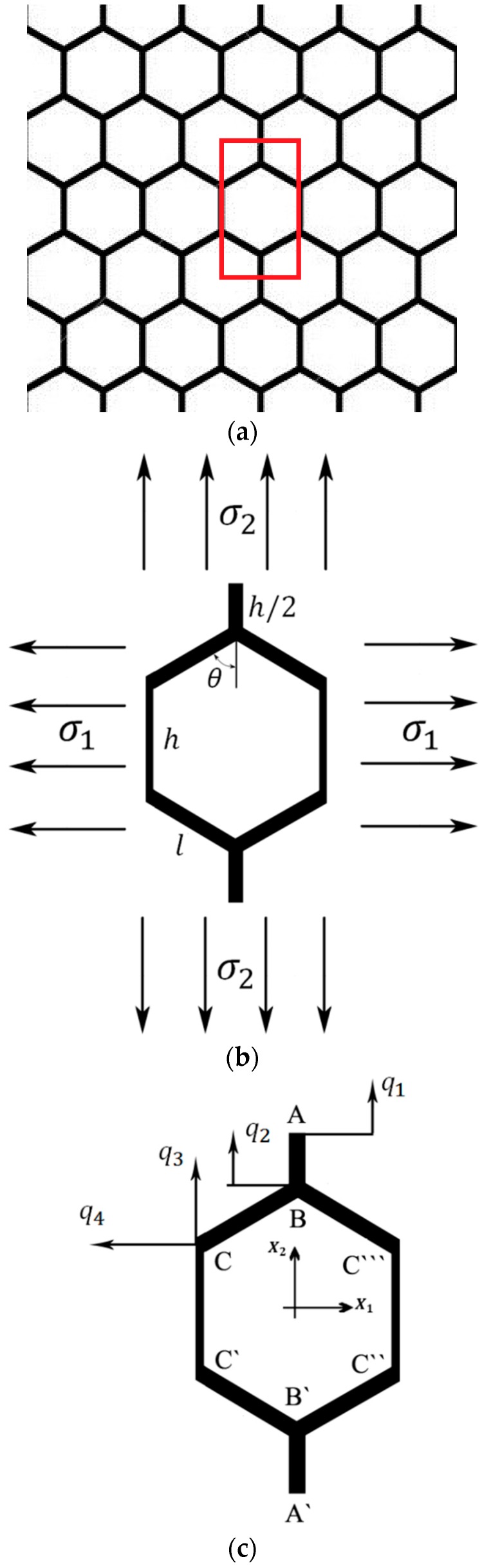
(**a**) A honeycomb structure; (**b**) a single unit cell of the honeycomb considered for analytical solution; and (**c**) degrees of freedom acting on the considered unit cell.

A unit cell (Figure 2b) constructing a 2D hexagonal lattice structure (Figure 2a) consists of four vertical and four inclined edges. The lengths of the vertical and inclined edges are assumed to be possibly different and are denoted by l and h, respectively. The angle between the inclined edges and the $X_{2}$ axis is also arbitrary and is denoted by θ. The thickness of the edges t is however considered constant throughout the study. It is customary in the studies investigating cellular solids to express the mechanical properties as functions of relative density (also called apparent density) rather than other geometrical parameters such as t/l. Dealing with relative density ($\frac{\rho }{\rho_{s}}$) rather than other geometrical parameters gives a better overview of the weight of the structure, and also makes it easier to compare the mechanical properties of structures with different micro-geometrical features but of the same weight.

**Figure 3:**
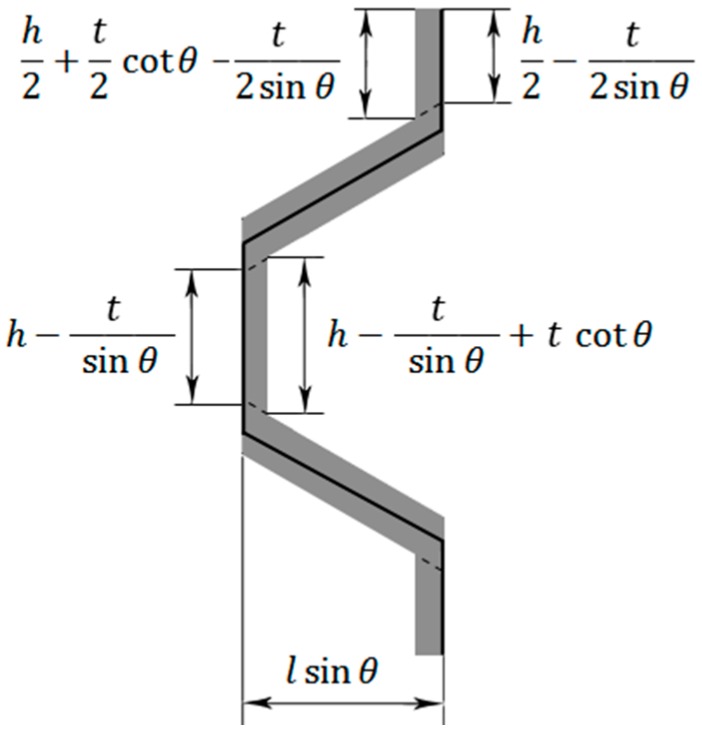
Dimensions of half a unit cell.

In thin honeycombs, i.e., when the ratio t/l is very small, the area occupied by the material constructing half a unit cell (Figure 3) is $2\left(\frac{h}{2}\frac{t}{2}+lt+\frac{h}{2}\frac{t}{2}\right)$. On the other hand, the total area occupied by half a unit cell is (2h+2lcos θ)l sinθ from which the relative density is given by

(1) $$\mu =\frac{\rho }{\rho_{s}}=\frac{t\left(h+2l\right)}{2\left(h+lcos\;\theta \right)l\;sin\theta }$$

In thick honeycombs, the area occupied by the material of half a unit cell is (see Figure 3)

(2) $$A=2\left[\frac{1}{2}\left(\frac{h}{2}-\frac{t}{2\sin\theta }+\frac{h}{2}+\frac{t}{2}\cot\theta -\frac{t}{2\sin\theta }\right)\frac{t}{2}+\frac{t}{\sin\theta }l\sin\theta \right]\;\;\;\;\;\;\;\;\;\;\;\;\;\;\;\;\;\;\;\;\;\;\;\;\;\;\;\;\;\;\;\;\;\;\;\;\;\;\;\;\;\;\;\;\;\;\;\;\;\;\;\;\;\;\;+\frac{1}{2}\frac{t}{2}\left(h-\frac{t}{\sin\theta }+h-\frac{t}{\sin\theta }+t\cot\theta \right)=th-\frac{t^{2}}{\sin\theta }+\frac{t^{2}}{2}\cot\theta +2\;tl$$

from which the relative density can be calculated as

(3) $$\mu =\frac{\rho }{\rho_{s}}=\frac{t\left(h+2l-\frac{t}{\sin\theta }+\frac{t}{2}\cot\theta \right)}{2\left(h+lcos\;\theta \right)l\;sin\theta }$$

**Figure 4:**
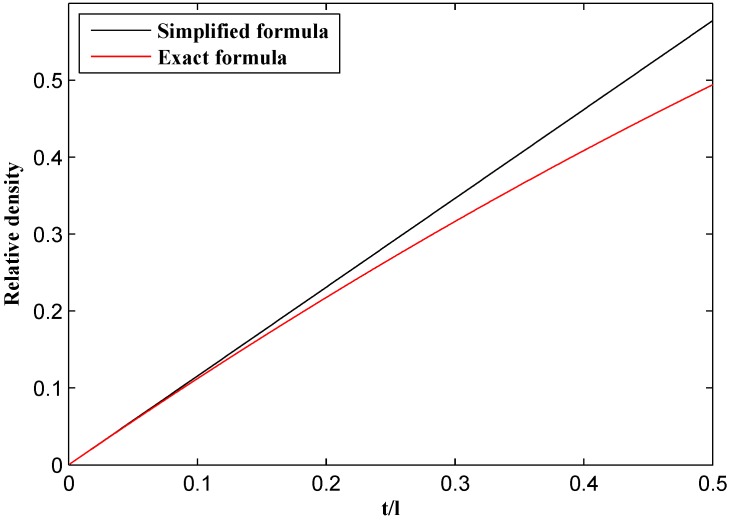
Comparison of relative density values based on simplified or exact formulas for regular hexagonal honeycombs.

Plotting the simplified and exact relative density relationships (i.e., Equations (1) and (3)) for regular hexagonal honeycombs showed that their values are close regardless of t/l (see Figure 4). Therefore, Equation (1) will be used from now on for calculating relative density because of its simplicity and also due to its use in the work by Gibson and Ashby [10,27].

### 2.3. Euler-Bernoulli Beam Theory

In this section, we use the Euler-Bernoulli beam theory to derive analytical relationships for the elastic modulus, E, Poisson’s ratio, ν, and yield stress, $\sigma_{y}$ of hexagonal honeycombs as functions of the elastic modulus, Poisson’s ratio, and yield stress of the bulk material ($E_{s},\;\sigma_{y_{s}},\;\nu_{s}$) and the relative density (μ) of the honeycomb structure. Since the in-plane deformation of the honeycombs is plane-strain, the problem is solved using beam elements with square cross-section.

The geometry and deformation of a honeycomb unit cell under simple axial loading is symmetrical with respect to both $X_{1}$ and $X_{2}$ directions. Therefore, the deformation of ¼ of a unit cell is representative of the deformation of all the four quarters. The symmetry of the unit cell with respect to $X_{1}$ and $X_{2}$ also implies that edges AB and A`B` (Figure 2c) remain straight during the elastic deformation and that they are only contracted or expanded with no additional flexure. Therefore, each of points A and B have only one degree of freedom denoted by $q_{1}$ and $q_{2}$, respectively. Since the deformation of edge CC` in a unit cell is symmetrical with respect to the deformation of edge C``C``` in the neighbouring unit cell located on its left side, similar to edge AB, edge CC` cannot have any rotation or lateral deflection along its length. Therefore, point C can have only two degrees of freedom, which are displacements in the $X_{1}$ and $X_{2}$ directions and are denoted $q_{4}$ and $q_{3}$, respectively. Edge BC cannot have any rotation at its ends B and C, but it can bend in its middle part.

As for the bending moment, the Euler–Bernoulli beam equation can be written as

(4) $$\frac{d^{4}w}{dx^{4}}=0$$

where w is the deflection of the mid-surface and x is the coordinate of the considered point. The solution to this differential equation can be expressed as:

(5) $$w=c_{0}+c_{1}x+c_{2}x^{2}+c_{3}x^{3}$$

where constants $c_{0}-c_{3}$ must be determined by applying certain boundary conditions. For a cantilever Euler-Bernoulli beam at the free end of which a point load F acts, we have

(6) $$\delta =\frac{Fl^{3}}{3E_{s}I}\;and\;\theta =\frac{Fl^{2}}{2E_{s}I}$$

and for a cantilever beam with a concentrated moment M at its end, the displacement and rotation are

(7) $$\delta =\frac{Ml^{2}}{2E_{s}I}\;and\;\theta =\frac{Fl}{E_{s}I}$$

In beams that the angle of the free end does not change during the deformation (e.g., the edges of the honeycomb considered in this study), the rotations produced by the lateral load F and moment M must be equal and opposite from which the value of M can be identified:

(8) $$\frac{Fl^{2}}{2E_{s}I}=\frac{Ml}{E_{s}I}\;\Rightarrow \;M=\frac{Fl}{2}$$

**Figure 5:**
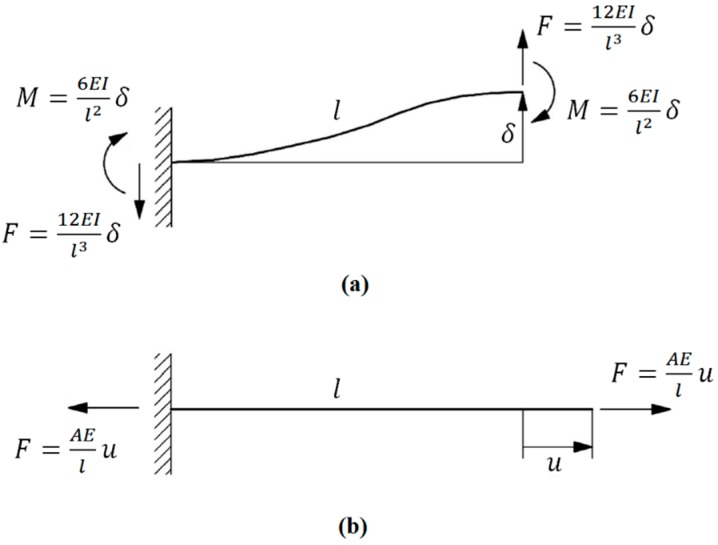
Forces and moments required to cause (**a**) lateral displacement δ with no rotation at the free end of a beam; and (**b**) simple axial expansion u.

While the force F tends to increase the lateral displacement, the moment M tends to reduce the deflection (Figure 5a). The total deflection resulted from force F and moment M is then

(9) $$\delta =\frac{Fl^{3}}{3E_{s}I}-\left(\frac{Fl}{2}\right)\frac{l^{2}}{2E_{s}I}=\frac{Fl^{3}}{12E_{s}I}$$

Rewriting Equation (9) as a function of F gives (see Figure 5a)

(10) $$F=\frac{12E_{s}I}{l^{3}}\;\delta$$

Similarly, the axial force required to displace the end of a rod by u is $AE_{s}u/l$ (see Figure 5). Figure 5 will be referred to several times in the rest of the paper for determining the forces and moments.

### 2.4. Timoshenko Beam Theory

The Timoshenko beam theory takes into account shear deformation and rotational inertia effects, making it suitable for describing the behavior of thick beams. For a homogenous beam of constant cross-section, the governing equations of the Timoshenko beam theory are:

(11) $$\frac{d^{2}}{dx^{2}}\left(E_{s}I\frac{d\varphi }{dx}\right)=q\left(x,t\right)\;\frac{dw}{dx}=\varphi -\frac{1}{\kappa AG_{s}}\frac{d}{dx}\left(E_{s}I\frac{d\varphi }{dx}\right)$$

where φ is the angle of rotation of the normal to the mid-surface of the beam and κ is the shear coefficient factor. The shear coefficient factor is $10\left(1+\nu_{s}\right)/\left(12+11\nu_{s}\right)$ for a rectangular cross-section (such as the beams considered in this study). In a linear elastic Timoshenko beam, the bending moment M is related to the angle of rotation φ by

(12) $$M=-E_{s}I\frac{\partial \varphi }{\partial x}$$

For a cantilever Timoshenko beam with a point load F at its free end, M=Fl and Equations (11) lead to

(13) $$\delta =\frac{Fl^{3}}{3E_{s}I}+\frac{Fl}{\kappa AG_{s}}\;and\;\theta =\frac{Fl^{2}}{2E_{s}I}+\frac{F}{\kappa AG_{s}}$$

The displacement and rotation of a cantilever Timoshenko beam with a concentrated moment M at its free end are identical to those of a similar Euler-Bernoulli beam. As before, the rotations produced by the lateral load F and moment M must be equal and opposite from which M can be calculated

(14) $$\frac{Fl^{2}}{2E_{s}I}+\frac{F}{\kappa AG_{s}}=\frac{F\frac{\sqrt{2}}{16}l^{2}}{2E_{s}I}=\frac{Ml}{E_{s}I}\;\Rightarrow \;M=F\left(\frac{l}{2}+\frac{E_{s}I}{\kappa AG_{s}l}\right)$$

While F tends to increase the lateral displacement, M tends to reduce it. The total deflection caused by F and M is then

(15) $$\delta =\frac{Fl^{3}}{3E_{s}I}+\frac{Fl}{\kappa AG_{s}}-\left(\frac{Fl}{2}+\frac{FE_{s}I}{\kappa AG_{s}l}\right)\frac{l^{2}}{2E_{s}I}=\frac{Fl^{3}}{12E_{s}I}+\frac{Fl}{2\kappa AG_{s}}$$

Rewriting Equation (15) as a function of F gives

(16) $$F=\frac{1}{\frac{l^{3}}{12E_{s}I}+\frac{l}{2\kappa AG_{s}}}\;\delta$$

### 2.5. Stiffness Matrix Derivation

Due to the symmetry of the hexagonal unit cell, ¼ of the unit cell was considered for the analytical study (the top left part of Figure 2c). Therefore, the obtained force at points A and B must be multiplied by two to calculate the total force applied to the corresponding degrees of freedom (DOFs) $q_{1}$ and $q_{2}$, respectively. Similarly, the obtained forces for point C must be multiplied by four to give the total force applied to the third and fourth DOFs, i.e., $q_{3}$ and $q_{4}$.

In this study, the stiffness matrix of the unit cell is obtained which is then used to calculate the displacements of the DOFs of the structure as functions of the imposed force. Given the displacements of the DOFs, analytical relationships for the mechanical properties of the unit cell can be derived. The displacements of a hexagonal unit cell can be considered as superposition of four distinct displacements taking place at each DOF separately. To obtain the elements of the ith column of the stiffness matrix, DOF $q_{i}$ is displaced by unity while the other DOFs are kept fixed. The forces that must be applied at each DOF to cause such a deformation constitute column i of the stiffness matrix. By applying this technique to all the DOFs, the elements of all the columns of the stiffness matrix are obtained. The force-displacement relationship of this system has the following form:

(17) $$\left\{\begin{array}{c} Q_{1}\\ Q_{2}\\ Q_{3}\\ Q_{4} \end{array}\right\}=\left[\begin{array}{cccc} k_{11} & k_{12} & k_{13} & k_{14}\\ k_{21} & k_{22} & k_{23} & k_{24}\\ k_{31} & k_{32} & k_{33} & k_{34}\\ k_{41} & k_{42} & k_{43} & k_{44} \end{array}\right]\left\{\begin{array}{c} q_{1}\\ q_{2}\\ q_{3}\\ q_{4} \end{array}\right\}$$

where $Q_{i}$ is the external force applied to a DOF $q_{i}$. In the following, the procedure of obtaining the stiffness matrix elements, $k_{ij}$, is presented. When applying the displacements, it is necessary to know what external forces must be applied at each DOF. Figure 5 demonstrates the loads required to cause lateral and axial unit displacements at the free end of a cantilever Euler-Bernoulli beam. This figure is referred to several times in the following.

#### 2.5.1. The First DOF: $q_{1}=1$

**Figure 6:**
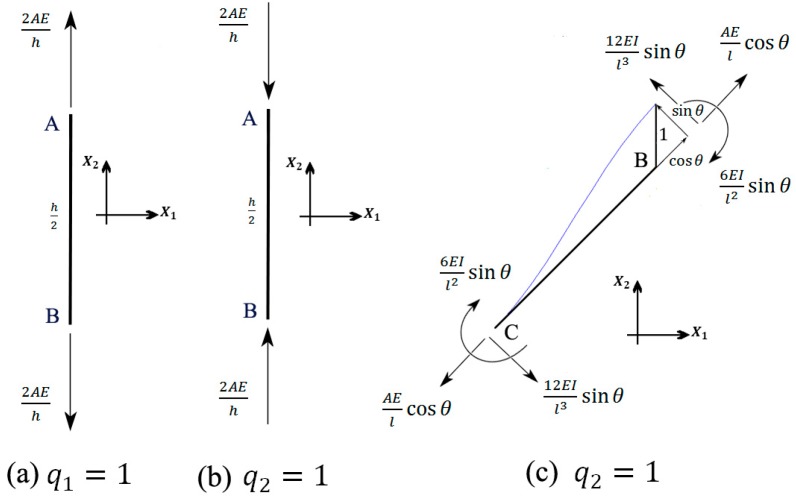
Free-body diagrams of struts (**a**) AB in deformation $q_{1}=1$; (**b**) AB in deformation $q_{2}=1$; and (**c**) BC in deformation $q_{2}=1$ of the hexagonal honeycomb unit cell.

In this subsection, the elements of the first column of the stiffness matrix are derived by applying the displacement $q_{1}=1$ and setting $q_{2}=q_{3}=q_{4}=0$. This deformation displaces the top and bottom points A and A` by unity upwards and downwards, respectively. Due to this deformation, strut AB is axially stretched by unity and applies the force $\frac{2{\text{AE}}_{s}}{h}$ (see Figure 6a) to points A and B. In order to have such a deformation, the forces $\frac{Q_{1}}{2}=\frac{2{\text{AE}}_{s}}{h}$ and $\frac{Q_{2}}{2}=-\frac{2{\text{AE}}_{s}}{h}$ must be applied to points A and B which give $Q_{1}=k_{11}=\frac{4{\text{AE}}_{s}}{h}$ and $Q_{2}=k_{21}=-\frac{4{\text{AE}}_{s}}{h}$. The negative value of $Q_{2}$ implies that in order to keep point B fixed, the external load in point B must be in the opposite direction of $q_{2}$. Since beams BC and CC` are not affected by this deformation mode, no external force is needed to be applied to point C, hence $Q_{3}=k_{31}=Q_{4}=k_{41}=0$.

#### 2.5.2. The Second DOF: $q_{2}=1$

In this case, beams AB and A`B` go under pure compression. In contrast to case $q_{1}=1$, here we have $\frac{Q_{1}}{2}=-\frac{2{\text{AE}}_{s}}{h}$ and $\frac{Q_{2}}{2}=\frac{2{\text{AE}}_{s}}{h}$ (Figure 6b). Unlike the previous case ($q_{1}=1$), here beam BC does deform (Figure 6c). The displacement of point B can be decomposed into two displacement of sinθ perpendicular to the undeformed beam BC and cosθ along it (Figure 6c). To have such axial and lateral displacements, the forces $\frac{{\text{AE}}_{s}}{l}\cos\theta$ and $\frac{12E_{s}I}{l^{3}}\sin\theta$ must have been applied to the ends of beam BC (Figure 6c). Equilibrium of forces in the $X_{1}$ direction at point B gives (Figure 6b,c)

(18) $$\sum^{\text{​}}f_{X1,\;B}=0\;\to \;-2\left(\frac{AE_{s}}{l}\cos^{2}\theta +\frac{12E_{s}I}{l^{3}}\sin^{2}\theta \right)-\frac{2AE_{s}}{h}+\frac{Q_{2}}{2}=0\;\to \;Q_{2}=k_{22}=4\left(\frac{AE_{s}}{l}\cos^{2}\theta +\frac{12E_{s}I}{l^{3}}\sin^{2}\theta \right)+\frac{4AE_{s}}{h}$$

Beam CC` is fixed and therefore imposes no forces to point C. Force equilibrium at point C in the $X_{1}$ and $X_{2}$ directions gives (Figure 6c)

(19) $$\sum^{\text{​}}f_{X1,\;C}=0\;\to \;-\left(\frac{AE_{s}}{l}\cos\theta \sin\theta +\frac{12E_{s}I}{l^{3}}\cos\theta \sin\theta \right)+\frac{Q_{4}}{4}=0\;\to \;Q_{4}=k_{42}=4\cos\theta \sin\theta \left(\frac{AE_{s}}{l}-\frac{48E_{s}I}{l^{3}}\right)$$

(20) $$\sum^{\text{​}}f_{X2,\;C}=0\;\to \;\frac{AE_{s}}{l}\cos^{2}\theta +\frac{12E_{s}I}{l^{3}}\sin^{2}\theta +\frac{Q_{3}}{2}=0\;\to \;Q_{3}=k_{32}=-4\frac{AE_{s}}{l}\cos^{2}\theta -\frac{48E_{s}I}{l^{3}}\sin^{2}\theta$$

#### 2.5.3. The Third DOF: $q_{3}=1$

**Figure 7:**
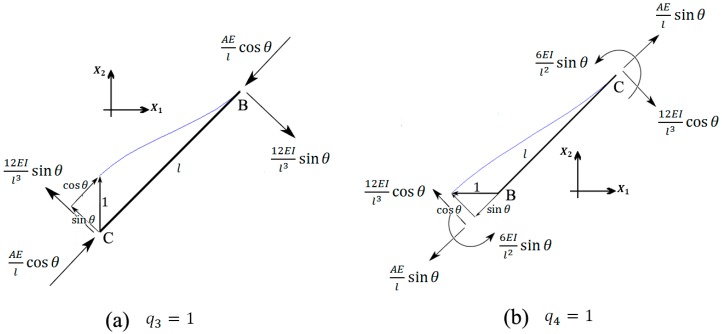
Free-body diagrams of strut BC in deformations (**a**) $q_{3}=1$; and (**b**) $q_{4}=1$.

This deformation type displaces vertex C upward by unity. Beam AB does not deform and, thus, does not impose any load to point B. Point A is not influenced by this deformation mode, thus $Q_{1}=k_{13}=0$. Force equilibrium at point B in the $X_{2}$ direction gives (Figure 7a)

(21) $$\sum^{\text{​}}f_{X2,\;B}=0\;\to \;2\left(\frac{AE_{s}}{l}\cos^{2}\theta +\frac{12E_{s}I}{l^{3}}\sin^{2}\theta \right)+\frac{Q_{2}}{2}=0\;\to \;Q_{2}=k_{23}=-4\left(\frac{AE_{s}}{l}\cos^{2}\theta +\frac{12E_{s}I}{l^{3}}\sin^{2}\theta \right)$$

Beam CC` (with length h and cross-section area of A/2) is stretched by 2 and therefore imposes the force ${\text{AE}}_{s}/h$ to point C. Force equilibrium at point C in the $X_{2}$ direction gives (Figure 7a)

(22) $$\sum^{\text{​}}f_{X2,\;C}=0\;\to \;-\frac{AE_{s}}{l}\cos^{2}\theta -\frac{12E_{s}I}{l^{3}}\sin^{2}\theta -\frac{AE_{s}}{h}+\frac{Q_{3}}{4}=0\;\to \;Q_{3}=k_{33}=\frac{4AE_{s}}{l}\cos^{2}\theta +\frac{48E_{s}I}{l^{3}}\sin^{2}\theta +\frac{4AE_{s}}{h}$$

Similarly, force equilibrium at the same point in the $X_{1}$ direction gives (Figure 7a)

(23) $$\sum^{\text{​}}f_{X1,\;C}=0\;\to \;\cos\theta \sin\theta \;\left(-\frac{AE_{s}}{l}+\frac{12E_{s}I}{l^{3}}\right)+\frac{Q_{4}}{4}=0\;\to \;Q_{4}=k_{43}=\cos\theta \sin\theta \left(\frac{48E_{s}I}{l^{3}}-\frac{4AE_{s}}{l}\right)$$

#### 2.5.4. The Fourth DOF: $q_{4}=1$

This deformation type displaces vertex C towards the left by unity. Similar to the case $q_{3}=1$, we have $Q_{1}=k_{14}=0$. Force equilibrium at point B and in the $X_{2}$ direction gives (Figure 7b)

(24) $$\sum^{\text{​}}f_{X2,\;B}=0\;\to \;-\frac{2AE_{s}}{l}\cos\theta \sin\theta +\frac{24E_{s}I}{l^{3}}\cos\theta \sin\theta +\frac{Q_{2}}{2}=0\;\to \;Q_{2}=k_{24}=\left(\frac{4AE_{s}}{l}-\frac{48E_{s}I}{l^{3}}\right)\cos\theta \sin\theta$$

Beam CC` simply displaces without any deformation, and therefore does not impose any load to point C. Force equilibrium in the $X_{1}$ direction at point C gives (Figure 7b)

(25) $$\sum^{\text{​}}f_{X2,\;C}=0\;\to \;-\frac{AE_{s}}{l}\sin^{2}\theta -\frac{12E_{s}I}{l^{3}}\cos^{2}\theta +\frac{Q_{4}}{4}=0\;\to \;Q_{4}=k_{44}=\frac{4AE_{s}}{l}\sin^{2}\theta +\frac{48E_{s}I}{l^{3}}\cos^{2}\theta$$

Similarly, force equilibrium at the same point in the $X_{2}$ direction gives (Figure 7b)

(26) $$\sum^{\text{​}}f_{X1,\;C}=0\;\to \;\cos\theta \sin\theta \;\left(\frac{AE_{s}}{l}-\frac{12E_{s}I}{l^{3}}\right)+\frac{Q_{3}}{4}=0\;\to \;Q_{3}=k_{34}=\cos\theta \sin\theta \left(\frac{48E_{s}I}{l^{3}}-\frac{4AE_{s}}{l}\right)$$

#### 2.5.5. The Stiffness Matrix

Using the obtained stiffness matrix elements, the force-displacement relationship based on Euler-Bernoulli beam theory in the matrix form is

(27) $$\left\{\begin{array}{c} Q_{1}\\ Q_{2}\\ Q_{3}\\ Q_{4} \end{array}\right\}=\left[\begin{array}{cccc} \frac{4AE_{s}}{h} & -\frac{4AE_{s}}{h} & 0 & 0\\ -\frac{4AE_{s}}{h} & \frac{4AE_{s}}{l}\cos^{2}\theta +\frac{48E_{s}I}{l^{3}}\sin^{2}\theta +\frac{4AE_{s}}{h} & -\frac{4AE_{s}}{l}\cos^{2}\theta -\frac{48E_{s}I}{l^{3}}\sin^{2}\theta & \left(\frac{4AE_{s}}{l}-\frac{48E_{s}I}{l^{3}}\right)\cos\theta \sin\theta \\ 0 & -\frac{4AE_{s}}{l}\cos^{2}\theta -\frac{48E_{s}I}{l^{3}}\sin^{2}\theta & \frac{48E_{s}I}{l^{3}}\sin^{2}\theta +\frac{4AE_{s}}{l}\cos^{2}\theta +\frac{4AE_{s}}{h} & \left(-\frac{4AE_{s}}{l}+\frac{48E_{s}I}{l^{3}}\right)\cos\theta \sin\theta \\ 0 & \frac{4AE_{s}}{l}\cos\theta \sin\theta -\frac{48E_{s}I}{l^{3}}\sin\theta \cos\theta & \left(\frac{48E_{s}I}{l^{3}}-\frac{4AE_{s}}{l}\right)\cos\theta \sin\theta & \frac{48E_{s}I}{l^{3}}\cos^{2}\theta +\frac{4AE_{s}}{l}\sin^{2}\theta \end{array}\right]\left\{\begin{array}{c} q_{1}\\ q_{2}\\ q_{3}\\ q_{4} \end{array}\right\}$$

Comparison of Equations (10) and (16) shows that the matrix-form force-displacement relationship for the Timoshenko beam theory can be obtained by replacing $\frac{12E_{s}I}{l^{3}}$ in Equation (27) by $\frac{1}{\frac{l^{3}}{12E_{s}I}+\frac{l}{2\kappa AG_{s}}}$ which yields

(28) $$\left\{\begin{array}{c} Q_{1}\\ Q_{2}\\ Q_{3}\\ Q_{4} \end{array}\right\}=\left[\begin{array}{cccc} \frac{4AE_{s}}{h} & -\frac{4AE_{s}}{h} & 0 & 0\\ -\frac{4AE_{s}}{h} & \frac{4AE_{s}}{l}\cos^{2}\theta +\frac{4}{\frac{l^{3}}{12E_{s}I}+\frac{l}{2\kappa AG_{s}}}\sin^{2}\theta +\frac{4AE_{s}}{h} & -\frac{4AE_{s}}{l}\cos^{2}\theta -\frac{4}{\frac{l^{3}}{12E_{s}I}+\frac{l}{2\kappa AG_{s}}}\sin^{2}\theta & \left(\frac{4AE_{s}}{l}-\frac{4}{\frac{l^{3}}{12E_{s}I}+\frac{l}{2\kappa AG_{s}}}\right)\cos\theta \sin\theta \\ 0 & -\frac{4AE_{s}}{l}\cos^{2}\theta -\frac{4}{\frac{l^{3}}{12E_{s}I}+\frac{l}{2\kappa AG_{s}}}\sin^{2}\theta & \frac{4}{\frac{l^{3}}{12E_{s}I}+\frac{l}{2\kappa AG_{s}}}\sin^{2}\theta +\frac{4AE_{s}}{l}\cos^{2}\theta +\frac{4AE_{s}}{h} & \left(-\frac{4AE_{s}}{l}+\frac{4}{\frac{l^{3}}{12E_{s}I}+\frac{l}{2\kappa AG_{s}}}\right)\cos\theta \sin\theta \\ 0 & \frac{4AE_{s}}{l}\cos\theta \sin\theta -\frac{4}{\frac{l^{3}}{12E_{s}I}+\frac{l}{2\kappa AG_{s}}}\sin\theta \cos\theta & \left(\frac{4}{\frac{l^{3}}{12E_{s}I}+\frac{l}{2\kappa AG_{s}}}-\frac{4AE_{s}}{l}\right)\cos\theta \sin\theta & \frac{4}{\frac{l^{3}}{12E_{s}I}+\frac{l}{2\kappa AG_{s}}}\cos^{2}\theta +\frac{4AE_{s}}{l}\sin^{2}\theta \end{array}\right]\left\{\begin{array}{c} q_{1}\\ q_{2}\\ q_{3}\\ q_{4} \end{array}\right\}$$

Since point B is an internal vertex, no external force is applied to it. The external force applied to point C in the $X_{2}$ direction is zero, thus $Q_{2}=Q_{3}=0$. If the stress acting on the structure in the $X_{1}$ direction is denoted by $\sigma_{x}$, using the geometrical relations, the force acting on point C in the $X_{1}$ direction can be obtained as $2\left(h+l\cos\theta \right)\sigma_{1}\;b$, where b is the thickness of the honeycomb structure in its out of plane direction. Similarly, the force acting on point A in the $X_{2}$ direction is calculated as $2l\sin\theta \;\sigma_{2}\;b$, where $\sigma_{2}$ is the stress acting on the structure in the $X_{2}$ direction. The force vector is therefore

(29) $$\left\{\begin{array}{c} Q_{1}\\ Q_{2}\\ Q_{3}\\ Q_{4} \end{array}\right\}=\left\{\begin{array}{c} 2\left[2l\sin\theta \sigma_{2}\;b\right]\\ 0\\ 0\\ 4\left[2\left(h+l\cos\theta \right)\sigma_{1}\;b\right] \end{array}\right\}=\left\{\begin{array}{c} 4l\sin\theta \sigma_{2}\;b\\ 0\\ 0\\ 8\left(h+l\cos\theta \right)\sigma_{1}\;b \end{array}\right\}$$

### 2.6. The Obtained Elastic Properties

For any deformation, the unknown displacements can be simply obtained by inverting the stiffness matrix given in Equations (27) or (28) and multiplying it by the force vector given in Equation (29). Using the obtained unknowns, it is possible to calculate the elastic modulus, Poisson’s ratio, and yield stress of the honeycomb structure as functions of the geometrical and material properties $E_{s},\;\sigma_{y_{s}},\;\nu_{s}$.

#### 2.6.1. Elastic Modulus

The elastic modulus in each direction is found by dividing the applied stress in that direction by the resulting strain in that direction, i.e., $E_{1}=\sigma_{1}/\varepsilon_{1}=\sigma_{1}\left(l\sin\theta \right)/q_{4}$ and $E_{2}=\sigma_{2}/\varepsilon_{2}=\sigma_{2}\left(h+l\cos\theta \right)/q_{1}$. Using the Euler-Bernoulli stiffness matrix, the relative elastic modulus in the $X_{1}$ direction is obtained as

(30) $${\left(\frac{E}{E_{s}}\right)}_{1}=\frac{t^{3}}{l^{3}}\frac{l\;sin\theta }{h\left(cos\theta +1\right)\left(\sin^{2}\theta {\left(\frac{t}{l}\right)}^{2\;}+\;\cos^{2}\theta \right)}$$

and using the Timoshenko stiffness matrix, the relative elastic modulus in the $X_{1}$ direction is obtained as

(31) $${\left(\frac{E}{E_{s}}\right)}_{1}=\frac{t^{3}}{l^{3}}\frac{\;l\;sin\theta }{h\left(cos\theta +1\right)\left({cos}^{2}\theta +\;0.2{\left(\frac{t}{l}\right)}^{2}{cos}^{2}\theta +{\left(\frac{t}{l}\right)}^{2}\;+\;1.1\;\nu_{s}{\left(\frac{t}{l}\right)}^{2}\;{cos}^{2}\theta \right)}$$

The relative elastic modulus in the $X_{2}$ direction for the Euler-Bernoulli beam theory is

(32) $${\left(\frac{E}{E_{s}}\right)}_{2}=\frac{t^{3}}{l^{3}}\frac{\frac{h}{l}\;+\;cos\theta }{sin\theta \left(\frac{2h}{l}{\left(\frac{t}{l}\right)}^{2\;}+\;{\left(\frac{t}{l}\right)}^{2\;}{cos}^{2}\theta \;+\;{sin}^{2}\theta \right)}$$

and for the Timoshenko beam theory is

(33) $${\left(\frac{E}{E_{s}}\right)}_{2}=\frac{t^{3}}{l^{3}}\;\frac{\frac{h}{l}\;+cos\theta }{sin\theta \left(\frac{2h}{l}{\left(\frac{t}{l}\right)}^{2}+{\left(\frac{t}{l}\right)}^{2\;}+{sin}^{2}\theta \right)+{sin}^{3}\theta \left(0.2{\left(\frac{t}{l}\right)}^{2}+1.1{\left(\frac{t}{l}\right)}^{2}\;\nu_{s}\right)}$$

#### 2.6.2. Poisson’s Ratio

The Poisson’s ratio can be obtained by dividing the two strains in the $X_{1}$ directions. For $\nu_{12}$, we have $\nu_{12}=\frac{\varepsilon_{2}}{\varepsilon_{1}}=\frac{q_{1}}{q_{4}}\;\frac{\text{l sin}\theta }{\left(h+\text{lcos}\theta \right)}$ for $\sigma_{1}\neq 0$ and $\sigma_{2}=0$. Using the Euler-Bernoulli force-displacement relationship (i.e., Equation (27)), the Poisson’s ratio $\nu_{12}$ is found as

(34) $$\nu_{12}=\frac{l{sin}^{2}\theta cos\theta \left(l^{2}-t^{2}\right)}{\left(t^{2}{sin}^{2}\theta +\;l^{2}{cos}^{2}\theta \right)\left(h+lcos\theta \right)}$$

and for Timoshenko beam theory, it is

(35) $$\nu_{12}=\frac{l{sin}^{2}\theta cos\theta \;\left(l^{2}+\;0.2\;t^{2}+\;1.1\;t^{2}\nu_{s}\right)}{\left(l^{2}{cos}^{2}\theta +\;0.2\;t^{2}{cos}^{2}\theta \;+\;t^{2}+\;1.1\;t^{2}{cos}^{2}\theta \nu_{s}\right)\left(h\;+\;lcos\theta \right)}$$

For $\nu_{21}$, we have $\nu_{21}=\frac{\varepsilon_{1}}{\varepsilon_{2}}=\frac{q_{4}}{q_{1}}\frac{\left(h+\text{lcos}\theta \right)}{\text{l sin}\theta }$ for $\sigma_{2}\neq 0$ and $\sigma_{1}=0$, which for the Euler-Bernoulli beam theory gives

(36) $$\nu_{21}=\frac{cos\theta sin\theta \left(l^{2}-\;t^{2}\right)\left(h\;+l\;cos\theta \right)}{sin\theta \left(2ht^{2}+\;t^{2}l{cos}^{2}\theta +\;l^{3}\sin^{2}\theta \right)}$$

and for the Timoshenko beam theory, it is

(37) $$\nu_{12}=\frac{cos\theta \;sin\theta \left(h\;+\;lcos\theta \right)\;\left(l^{2}\;+\;0.2\;t^{2}\;+\;1.1\;t^{2}\nu_{s}\right)}{sin\theta \;\left(l^{3}\sin^{2}\theta +2\;t^{2}\;h+t^{2}\;l+\;0.2\;t^{2}l\;sin^{2}\theta +1.1\;t^{2}\;l\;\nu_{s}\sin^{2}\theta \right)}$$

#### 2.6.3. Yield Stress

In the FE simulations, it was seen that that the end points of the inclined edges are the location with maximum stress for all cases of axial loading in the $X_{1}$ direction, axial loading in the $X_{2}$ direction, and bi-axial loading. In a general deformation of beam BC, in which point B is dislocated by $q_{2}$ in the $X_{2}$ direction and point C is dislocated by $q_{4}$ and $q_{3}$ respectively in the $X_{1}$ and $X_{2}$ directions, by assuming that beam BC is clamped at one of its ends B or C, increase in the length of the beam BC is $q_{4}\sin\theta +\left(q_{2}-q_{3}\right)\cos\theta$. Similarly, the relative lateral displacement of the free end of beam BC is $\left(q_{2}-q_{3}\right)\sin\theta -q_{4}\cos\theta$. These displacements cause axial load (Figure 5b) and bending moments (Figure 5a) in the beam

(38) $$P=\frac{AE_{s}}{l}\left(q_{4}\sin\theta +\left(q_{2}-q_{3}\right)\cos\theta \right)\;M=\frac{6E_{s}I}{l^{2}}\left(\left(q_{2}-q_{3}\right)\sin\theta -q_{4}\cos\theta \right)$$

which impose the axial and flexural stresses of

(39) $$\sigma_{axial}=\frac{E_{s}}{l}\left(q_{4}\sin\theta +\left(q_{2}-q_{3}\right)\cos\theta \right)\;\sigma_{flexure}=\frac{3E_{s}t}{l^{2}}\left(\left(q_{2}-q_{3}\right)\sin\theta -q_{4}\cos\theta \right)$$

By adding the axial and flexural stresses given in the above equation, the maximum stress in the honeycomb unit cell can be found. The yield stress of the structure is then given by

(40) $$\sigma_{y}=\frac{\sigma_{ys}\sigma_{i}}{\sigma_{max}}$$

where $\sigma_{ys}$ is the yield stress of the bulk material, $\sigma_{i}$ is the applied stress in direction i, and $\sigma_{max}$ is the resulting maximum stress $\sigma_{max}=\sigma_{axial}+\sigma_{flexure}$. The relative yield stress in the $X_{1}$ direction for the Euler-Bernoulli beam theory is found as

(41) $${\left(\frac{\sigma_{y}}{\sigma_{ys}}\right)}_{1}=\frac{1}{h\;\left(1+\cos\theta \right)}\left(\frac{t^{2}}{t\;sin\theta +3l\;\cos\theta }\right)$$

The analytical relationship for the yield stress based on the Timoshenko beam theory was lengthy and, had limited influence on the yield stress. We therefore do not present the analytical relationship for the yield stress in the $X_{1}$ direction based on the Timoshenko beam theory. The relative yield stress in the $X_{2}$ direction was found as

(42) $${\left(\frac{\sigma_{y}}{\sigma_{ys}}\right)}_{2}=\frac{1}{l\;\sin\theta }\left(\frac{t^{2}}{3l\;\sin\theta -t\;\cos\theta }\right)$$

for the Euler-Bernoulli beam theory and

(43) $${\left(\frac{\sigma_{y}}{\sigma_{ys}}\right)}_{2}=\frac{1}{l\;\sin\theta }\left(\frac{t^{2}}{3l\;\sin\theta -t\cos\theta +3\frac{t^{2}}{l}\left(1.2+1.1\nu_{s}\right)\sin\theta }\right)$$

for the Timoshenko beam theory.

### 2.7. Computational Modelling

In this study, FE simulations were used as a validation tool for the analytical relationships derived above. The planar deformation of the honeycomb structures suggests using beam elements for representing the cell edges. All the links in the hexagonal honeycomb structure were represented mechanically by beams that were rigidly connected at the vertices. The edges were discretized using the standard Timoshenko beam elements that uses linear interpolation approximation and allows for transverse shear deformation. Considering transverse shear deformation becomes more important in thick beams (such as the ones constructing a high density honeycomb) compared to slender beams. Since in this study, the mechanical properties of the honeycomb are obtained in elastic regime, and since the results are reported in normalized values, the type of material for the FE modelling does not affect the results (i.e., the normalized values of mechanical properties). The material considered for the numerical analysis was steel and its mechanical behavior was assumed to be linear elastic, with the elastic modulus $E_{s}\;=\;200\;GPa$ and the Poisson’s ratio $\nu_{s}\;=\;0.3$.

**Figure 8:**
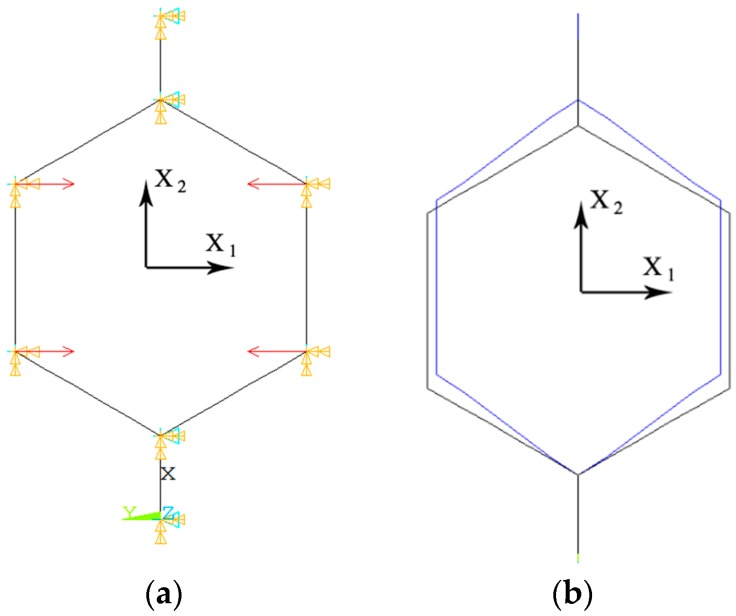
(**a**) The geometry, loads, and boundary conditions used in the 2D hexagonal honeycomb finite element (FE) model; and (**b**) its deformed shape, for $\sigma_{1}\neq 0$ and $\sigma_{2}=0$.

The static nonlinear implicit solver of ANSYS FE code was used for solving the problem. The geometry of the FE model (Figure 8) was identical to the geometry of the unit cell used for the analytical derivations (Figure 2). All the vertices were constrained in the $X_{3}$ direction (perpendicular to the page). The vertices A, B, A`, and B` connected to the two vertical beams AB and A`B` were constrained in the $X_{1}$ direction (see Figure 2). In order to avoid singularity of the pivot terms in the ANSYS solver caused by rigid body movements, the degrees of freedom of one of the vertices must be completely constrained in the space. Since this structure has no central vertex, the bottom point A` was fixed in space.

The elastic modulus of the structure in each direction was calculated by applying a uniaxial stress $\sigma_{i}$ and measuring the resulting strain in the same direction $\varepsilon_{i}$ and then dividing both values, i.e., $E_{i}=\frac{\sigma_{i}}{\varepsilon_{i}}$. The Poisson’s ratios were determined by dividing the negative value of the lateral strain by the axial strain. The yield stress was found by finding the maximum stress, $\sigma_{max}$, in the FE model and then substituting it in Equation (40).

## 3. Results

**Figure 9:**
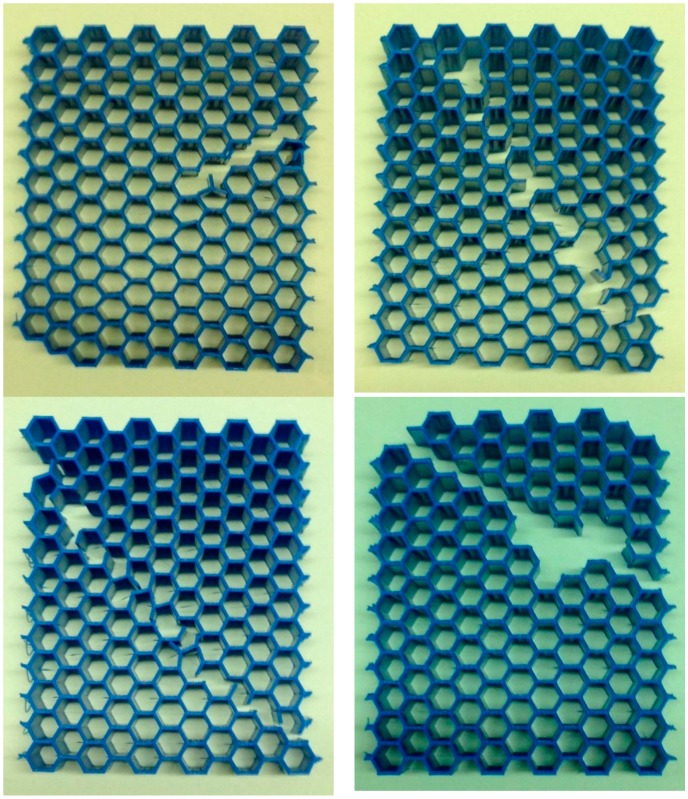
The 45° failure pattern in the honeycombs.

**Figure 10:**
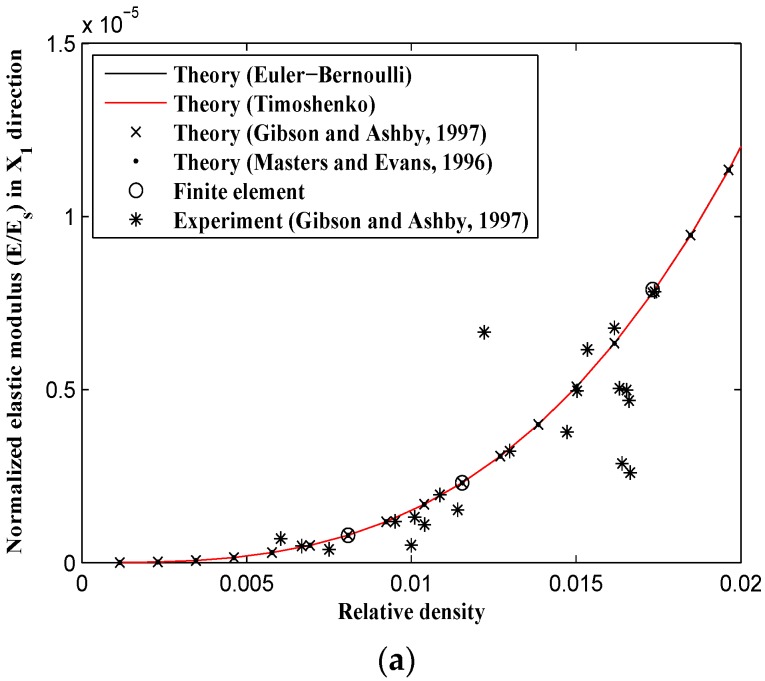

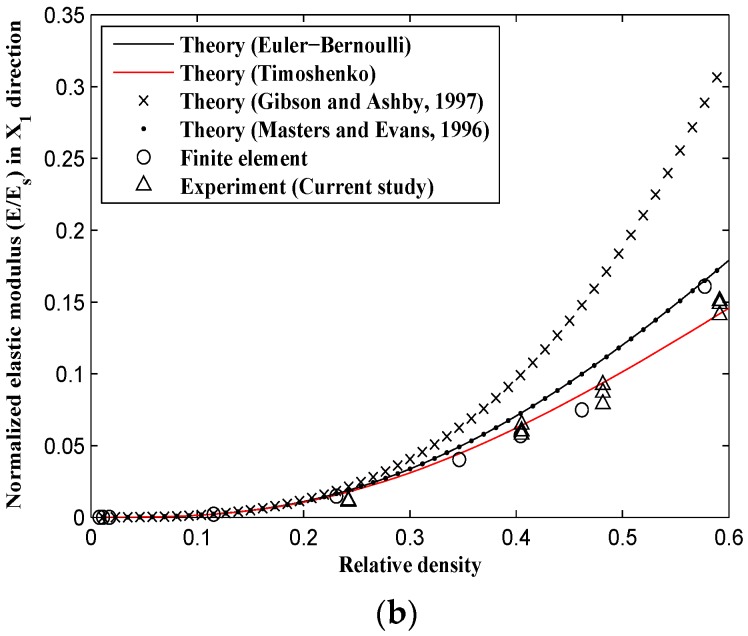
Variation of relative elastic modulus in $X_{1}$ direction ($E_{1}/E_{s}$) vs. relative density ($\rho /\rho_{s}$) for analytical, experimental, and numerical results. (**a**) For small relative density range; and (**b**) for the complete range of relative density.

**Figure 11:**
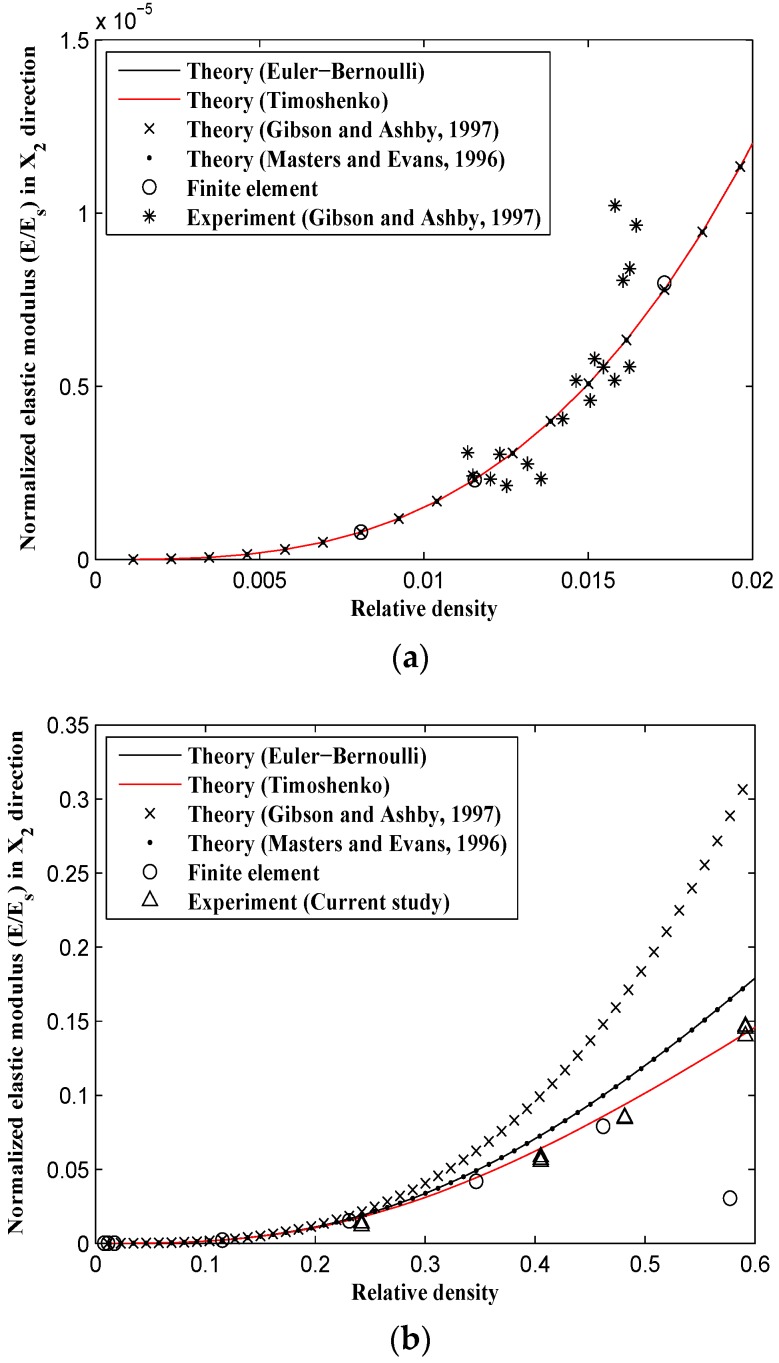
Variation of relative elastic modulus in $X_{2}$ direction ($E_{2}/E_{s}$) vs. relative density ($\rho /\rho_{s}$) for analytical, experimental, and numerical results. (**a**) For small relative density range; and (**b**) for the complete range of relative density.

All the samples showed 45° failure bands during their post yielding behavior (Figure 9). Since the experimental data provided by Gibson and Ashby [10,27] are only presented for very small relative densities (μ<0.02) and the experimental results obtained in this paper cover relatively large relative densities (0.2<μ<0.55), the diagram of each mechanical behavior is plotted in two ranges of relative densities: one from 0 to 0.02, and the other from 0 to 0.5. At very small relative densities, the elastic modulus obtained from the derived analytical formulas (based on both Timoshenko and Euler-Bernoulli beam theories), the analytical formulas presented by Gibson and Ashby [10,27] and Masters [22], the FE model, and Gibson and Ashby [10,27] experimental observations all coincide well with each other (Figure 10a and Figure 11a), but they start to deviate from each other as the relative density of the structure increases. For a relative density of 0.5, the elastic modulus predicted by the Gibson and Ashby formula is almost twice that obtained from the analytical Timoshenko formula (i.e., Equations (31) and (33)) and the FE model (Figure 10b and Figure 11b).

For large relative densities, the analytical elastic modulus formulas presented by Gibson and Ashby deviate significantly from the other results, i.e., the Euler-Bernoulli beam theory (obtained in this study and obtained by Masters and Evans [22]), the Timoshenko beam theory, the numerical results, and the experimental data of the tests carried out in this study (Figure 10b and Figure 11b). The elastic modulus formulas presented by Masters and Evans [22] and the Euler-Bernoulli-based formulas obtained in this study rely on each other for all the values of relative density. Moreover, the elastic modulus obtained based on the Timoshenko beam theory is smaller than that based on the Euler-Bernoulli beam theory and is generally in better agreement with numerical results (Figure 10b and Figure 11b). Compared to Gibson and Ashby analytical formulas and the derived Euler-Bernoulli theory, the Timoshenko analytical elastic modulus presented in this study corresponds much better with both experimental and numerical results.

**Figure 12:**
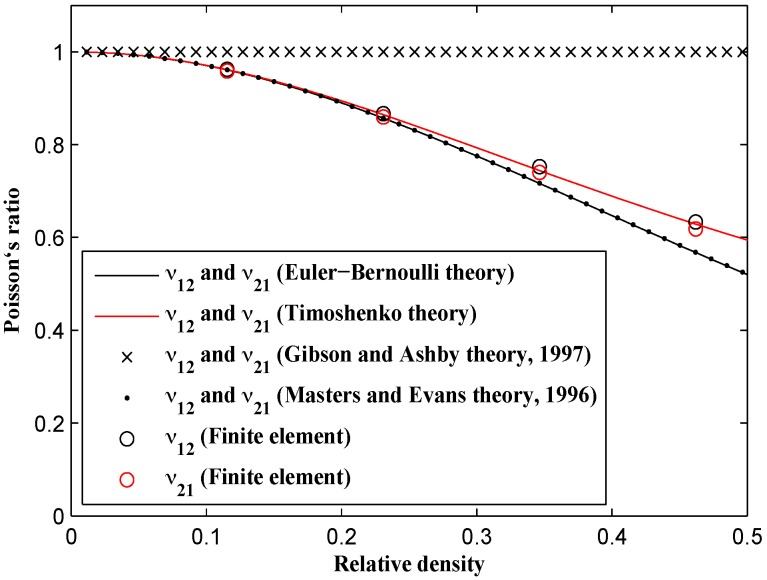
Comparison of analytical and numerical values of Poisson’s ratio for different relative densities.

The formulas presented by Gibson and Ashby predict a constant Poisson’s ratio (i.e., ν=1) for all values of relative density. Both the analytical formulas derived in this paper and our FE results, coincide with the Gibson and Ashby result at very small relative densities, but start to decrease as the relative density increases (Figure 12). The FE results almost coincide with the analytical results obtained using the Timoshenko beam theory. Moreover, the Poisson’s ratio formulas presented by Masters [22] and the Euler-Bernoulli-based formulas derived here lie on top of each other. The Poisson’s ratio value is identical in the $X_{1}$ and $X_{2}$ directions for both numerical and analytical results ($\nu_{12}=\nu_{21}$). At μ=0.5, the predicted Poisson’s ratios obtained from all the methods implemented in this study are all between 0.5 and 0.6, which is in contrast with the prediction of Gibson and Ashby theory which is 1. The experimental results obtained from a number of tests carried out by Gibson and Ashby [10,27] also show much smaller Poisson’s ratio than their theoretical predictions.

**Figure 13:**
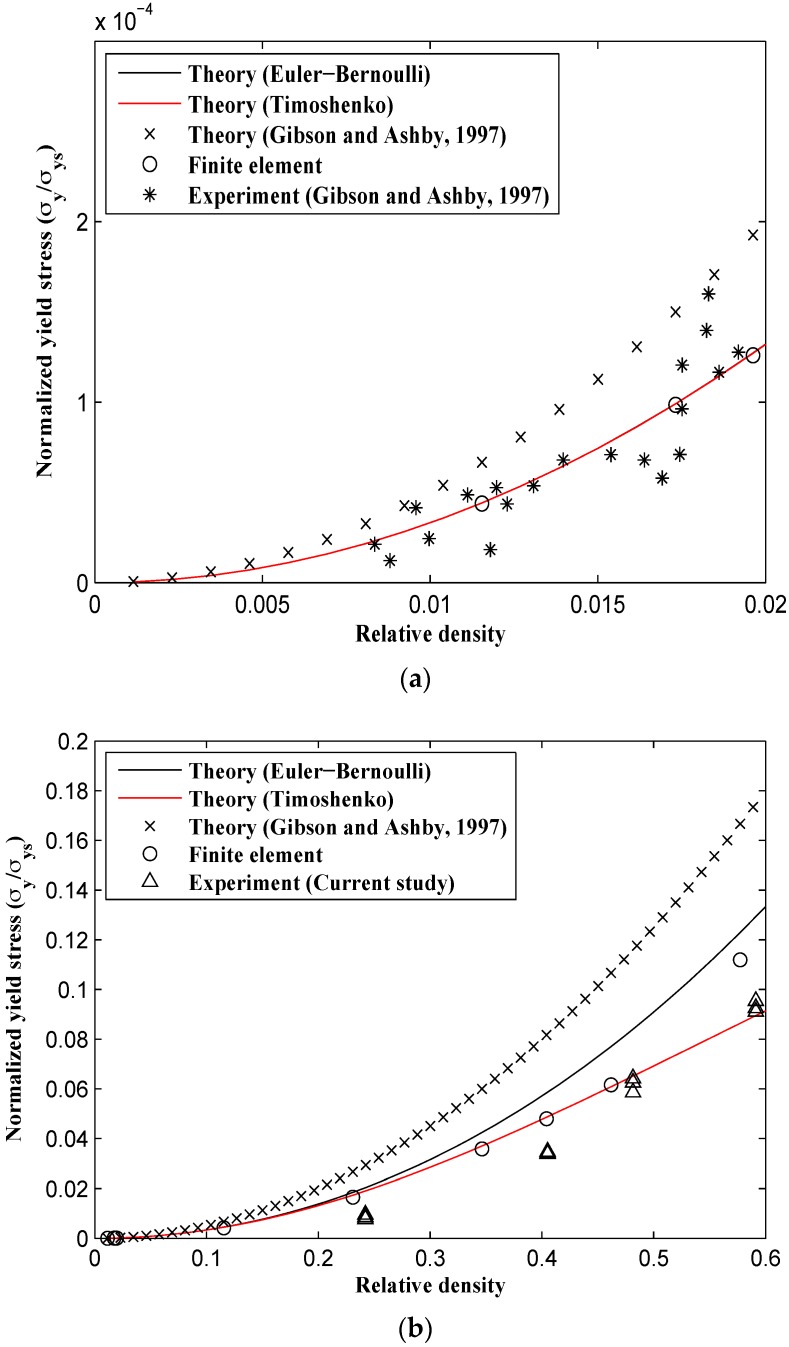
Variation of relative yield stress in $X_{1}$ direction ${\left(\sigma_{y}/\sigma_{ys}\right)}_{1}$ vs. relative density ($\rho /\rho_{s}$) for analytical, experimental, and numerical results. (**a**) For small relative density range; and (**b**) for the complete range of relative density.

**Figure 14:**
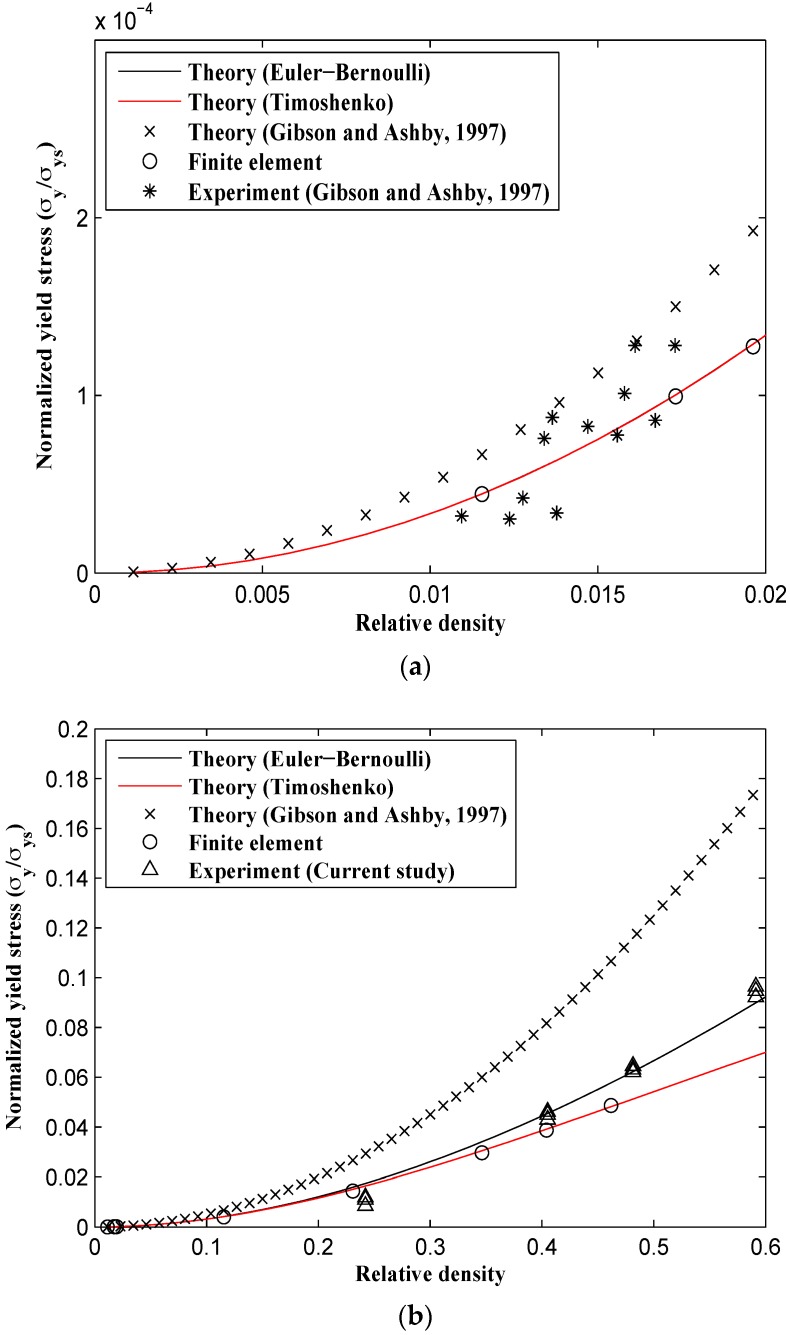
Variation of relative yield stress in $X_{2}$ direction ${\left(\sigma_{y}/\sigma_{ys}\right)}_{2}$ vs. relative density ($\rho /\rho_{s}$) for analytical, experimental, and numerical results. (**a**) for small relative density range; and (**b**) for the complete range of relative density.

Unlike the elastic modulus and Poisson’s ratio for which all the numerical and analytical methods gave very close results at small relative densities, the analytical formulas given by Gibson and Ashby predict different yield stresses even at small relative densities (Figure 13). The analytical formulas obtained in this study, the FE model, and Gibson and Ashby’s experimental data are in good agreement for small relative densities, but the analytical formulas presented by Gibson and Ashby are somewhat different from all other results (Figure 13a and Figure 14a). For example, for a small relative density of 0.02, the yield stress predicted by Gibson and Ashby formulas is about 30% higher than those predicted by other techniques. This deviation continues to increase for larger relative densities, especially in the $X_{2}$ direction (Figure 14b). At the relative density of 0.5, the yield stress $\sigma_{y2}$ predicted analytically by the relationships presented in the Gibson and Ashby study is at least twice that given by other techniques. The analytical relationships derived using both Euler-Bernoulli and Timoshenko theories almost coincide for relative densities smaller than 0.15 (Figure 13 and Figure 14). For all relative densities, the yield stress formula based on the Timoshenko beam theory correlates well with the experimental tests carried out in this study, Gibson and Ashby’s experimental results, and the FE results (Figure 13).

It is noteworthy to mention that the analytical relationships derived for the Poisson’s ratio and elastic modulus are identical in both major directions $X_{1}$ and $X_{2}$ (see Figure 12 and compare Figure 10 and Figure 11). Therefore, the reciprocal relationship $E_{1}\nu_{21}=E_{2}\nu_{12}$ is also valid for the honeycomb. However, the structure shows a higher yield stress in the $X_{1}$ direction. For example at μ=0.5, the yield stress in the $X_{1}$ direction is 18.5% higher than that in the $X_{2}$ direction. This large difference in the yield stress in both major directions disappears for small values of relative density (compare Figure 13a, Figure 14a). The yield stress formulas derived by Gibson and Ashby, however, predict similar yield stress values for both major directions.

## 4. Discussion

Unlike the 2D nature of deformation in honeycomb structures, the deformation of foam struts (or walls) can be under the effect of many different loading conditions such as torsion and bending in multiple directions. In honeycomb structures, due to the intrinsic simplicity and symmetry of cell geometries, the degrees of freedom of the structure are small. However, the freedom of the struts in foam structures to move in any direction and angle makes it much more difficult to obtain analytical relationships for such structures. In addition to the benefits stated before, studying the mechanical behavior of 2D honeycomb structures under in-plane loading has also the advantage that its results shed light on studying the much more complex responses of 3D tessellated structures, such as foams [27] and additively manufactured porous biomaterials [28,29,30]. The new matrix-based derivation of analytical relationships is very advantageous in simplifying very complex 3D unit cells with large degrees of freedom.

Honeycombs are usually constructed using the two manufacturing methods namely corrugation and HOBE (HOneycomb Before Expansion). Production of honeycombs using these two methods requires expensive equipment and several preparation methods. The advent of additive manufacturing (AM) techniques, such as selective laser melting (SLM) [31], selective electron beam melting (SEBM) [32], and selective laser sintering (SLS) [33], has enabled the production of several structures with complex geometries with remarkable ease. Porous structures with controllable unit cell type and size are among the many different structures that are currently being created using additive manufacturing methods. In recent years, the most focus has been on production and analysis of 3D structures with different unit cell geometries for biomedical applications, such as diamond [34,35], rhombic dodecahedron [36], truncated cuboctahedron [2], rhombicuboctahedron [37], truncated cube [3], etc. Production of honeycombs using additive manufacturing techniques [38,39] has the advantage of providing freedom in choosing the unit cell type. These techniques are also able to produce lattice structures with unit cell sizes smaller than 100 µm. The cell walls of the additively manufactured hexagonal honeycombs can be chosen to be thicker than traditional honeycombs (and in fact for cases with small unit cells, they have to be thick).

In denser honeycombs, the established in-plane analytical elastic modulus and Poisson’s ratio relationships derived by Gibson and Ashby [10] and Masters and Evans [22] show significant deviations from numerical and experimental results. In those cases, the analytical results obtained in this paper show much more accurate results. For small relative densities where the thickness of the cell walls are small compared to their length, neglecting the shear deformation and axial compression or tension of the cell walls does not have a negative effect on the prediction of the deformation of the geometry. In fact, in thin honeycombs, the beam is much weaker in the lateral direction as compared to the axial direction. Therefore, the axial compression or tension of the beam does not contribute considerably to the total deformation of the beam. However, as the relative density is increased, the flexural stiffness of the beams increases faster than the axial stiffness of the beam until it reaches a value that is comparable with the axial stiffness. In the analytical analysis carried out by Gibson and Ashby [10], the shear deformation and axial tension or compression of the beams are neglected, which explains the large discrepancy of elastic modulus and Poisson’s ratio in large t/l ratios.

Unlike the elastic modulus and Poisson’s ratio, the analytical yield stress formulae obtained by Gibson and Ashby [10] shows deviation from numerical and experimental results even at small relative densities. Our analytical yield stress values, however, show good correlation in all the relative density ranges. This discrepancy can also be explained by the terms neglected in Gibson and Ashby [10] derivations. While the structure elastic modulus and Poisson’s ratio values relate to the deformation of the beams, the structure yield stress relates to the stress generated in the beams. In small relative densities, the maximum stress resulted from the axial internal loads in the beams can also be high (since the generated axial stress in the beam is simply a result of the axial component of the applied external load applied to the beam), although the resulting deformation can be tiny (due to the much higher axial stiffness of the beam compared to their flexural stiffness). That is why the yield stress formula derived by Gibson and Ashby [10] shows considerable deviation from the other results (numerical, experimental, and the analytical formulas obtained in this paper) even at small t/l ratios.

Generally, there are two numerical approaches to model the honeycombs: macro-geometrical and micro-geometrical. In the micro-geometrical approach, all the cell walls are created in the FE model. This method is usually useful only for parts with not a very large number of cells. Since each wall has to be discretized using several elements, using the micro-geometrical approach for large parts is computationally expensive. In the macro-geometrical approach, the microstructure of the honeycomb is not modelled and simple cubic or square elements with an assigned honeycomb material model are implemented. Before creating a macro-geometrical FE model, knowing the effective elastic properties of the honeycomb is necessary [11] which is usually obtained from experimental tests. In both the numerical modelling methods, the user has to handle modelling parameters for achieving accurate results that sometimes are complex to deal with. Compared to numerical modelling, understanding the mechanism and physical effects through the problem is much easier and faster using analytical relationships [40].

The proposed methodology is quite general and applies to additive manufactured and conventionally manufactured honeycombs alike. The use of additive manufacturing techniques is, however, important from two viewpoints. First, different designs of honeycombs with different shapes could be easily realized with additive manufacturing techniques. Since the essence of the methodology proposed here is applicable to other geometries, we think the fundamental aspects of the proposed analytical techniques could be used for a wide range of additively manufactured honeycombs perhaps with some modifications in some of the derivation steps of the analytical relationships. Moreover, additive manufacturing techniques could be used for designing more complex geometrical shapes in general and gradients in the wall thickness and pore geometry in particular. This form-freedom creates various design opportunities that could be best utilized when the effects of changes in the design of the honeycombs on the mechanical behavior of the resulting scaffolds could be easily predicted. The analytical relationships presented here and their variants could be used to predict the mechanical properties of the honeycombs resulting from various design options.

## 5. Conclusions

The main contribution of this research was the derivation of analytical relationships for elastic properties (elastic modulus, Poisson’s ratio, and yield stress) of hexagonal honeycomb structures in their two major in-plane directions. Towards this end, the stiffness matrices of a hexagonal honeycomb unit cell were obtained using both Euler-Bernoulli and Timoshenko beam theories. An FE model was also created for validation of the proposed analytical relationships as well as to illustrate the required steps required for development of a trustworthy numerical tool for investigation of plane-strain honeycomb structures. Several structures were also manufactured using a filament-based additive manufacturing machine. Compared to the existing analytical relationships for in-plane deformation of hexagonal honeycombs presented by Gibson and Ashby [10] and Masters and Evans [22], the obtained analytical relationships in this study for both Euler-Bernoulli and Timoshenko beam theories were much closer to the experimental and numerical results.
